# Incidence proportions and prognosis of breast cancer patients with bone metastases at initial diagnosis

**DOI:** 10.1002/cam4.1668

**Published:** 2018-07-09

**Authors:** Yue Gong, Jing Zhang, Peng Ji, Hong Ling, Xin Hu, Zhi‐Ming Shao

**Affiliations:** ^1^ Department of Breast Surgery Key Laboratory of Breast Cancer in Shanghai Fudan University Shanghai Cancer Center Fudan University Shanghai China; ^2^ Department of Oncology Shanghai Medical College Fudan University Shanghai China; ^3^ Department of Orthopedics Shanghai Jiao Tong University Affiliated Sixth People's Hospital Shanghai China; ^4^ Institutes of Biomedical Science Fudan University Shanghai China

**Keywords:** bone metastases, breast cancer, incidence, prognosis

## Abstract

**Introduction:**

Population‐based data on the incidence and prognosis of bone metastases at diagnosis of breast cancer are currently limited. Hence, we conducted this study to analyze the incidence proportions and prognostic factors of patients with breast cancer and bone metastases at the time of cancer diagnosis.

**Materials and methods:**

Patients with primary invasive breast cancer and bone metastases at initial diagnosis between 2010 and 2014 were identified using the Surveillance, Epidemiology, and End Results (SEER) dataset and Fudan University Shanghai Cancer Center (FUSCC) cohort. Multivariable logistic regression was performed to identify predictors of the presence of bone metastases at diagnosis. Univariate and multivariate analyses were performed to determine the effects of each variable on survival.

**Results:**

Of 229, 195 patients from SEER database included in the analysis, 8295 patients had bone metastases at initial diagnosis, reflecting 3.6% of the entire study population, and 65.1% of the subset with metastatic disease to any distant site. Patients with hormone receptor (HR)‐positive human epidermal growth factor receptor 2 (HER2)‐negative represented the highest incidence proportions among patients with metastatic disease (73.9%). Among entire cohort, multivariable logistic regression identified eight factors as predictors of the presence of bone metastases at diagnosis. Median OS for the patients with bone metastases in SEER and FUSCC cohorts was 30.0 and 68.2 months, respectively. Patients with HR‐positive HER2‐positive subtype had the longest median OS, and patients with triple‐negative subtype showed the shortest median OS. Multivariable Cox model in SEER cohort confirmed age, histology, grade, tumor subtype, extraosseous metastatic sites, history of primary surgery, insurance status, marital status, and income as independent prognostic factors for both OS and BCSS.

**Conclusions:**

The findings of this study provide population‐based estimates of the incidence and prognosis for patients with bone metastases at initial diagnosis of breast cancer.

## 1 INTRODUCTION

Breast cancer is the most common cancer in women and is the leading causes of cancer‐related death in women. Globally, it accounted for approximately 1.67 million cases in 2012 and 0.52 million deaths, and both numbers have continued to increase.^1^ Approximately 5% of patients present with distant metastases at their initial diagnosis of breast cancer, with bone being the most common site.^2^, ^3^ Of those patients who die of breast cancer, approximately 70% will have evidence of bone metastases.^4^

Bone metastases are associated with lower survival in patients with advanced breast cancer and an increased risk of serious complications during the patients’ disease course.^5^, ^6^, ^7^ The sites and extent of metastases determine the complications, which are called skeletal‐related events (SREs); these include pathological fractures, severe bone pain, bone marrow infiltration, spinal cord compression, and hypercalcemia.^8^, ^9^ The median time from bone metastases diagnosis to first SRE among breast cancer patients with bone metastases is only 1.8 months, and the 1‐year incidence of SREs is as high as 40%.^2^ Studies have demonstrated that nearly 50% of breast cancer patients with bone metastases experience at least one of these SREs, resulting in the reduced quality of life.^2^, ^7^, ^10^

There is growing evidence indicating that patterns of metastases vary between different hormone receptor (HR) and human epithelial growth factor receptor 2 (HER2) statuses of breast tumors.^11^, ^12^, ^13^, ^14^, ^15^, ^16^, ^17^, ^18^ Patients with HR‐positive breast cancer are at increased risk for the development of bone metastases, whereas bone metastases are less frequent in cases of triple‐negative tumors.^15^, ^16^, ^17^, ^18^, ^19^, ^20^, ^21^ Previous studies also indicated that tumor subtype is an important prognostic factor for the survival of patients with bone metastases, where worse survival was seen in patients with triple‐negative breast cancer.^22^, ^23^

An early diagnosis of bone metastases is necessary to start intervention early and reduce complications. However, current guidelines do not recommend routine screening of bone metastases in patients with localized breast cancer only if directed by signs or symptoms.^24^ Among patients with bone metastases at initial diagnosis of breast cancer, there is a lack of data regarding patient characteristics and the clinical and sociodemographic predictors of outcome at a population level, which needs to be supplemented and studied.

In this study, we used the Surveillance, Epidemiology, and End Results (SEER) database to survey the incidence of bone metastases when breast cancer was initially diagnosed. We also attempted to investigate the influence of tumor subtype and other prognostic factors on the survival of patients with bone metastases at the time of cancer diagnosis using both SEER cohort and another independent cohort from Fudan University Shanghai Cancer Center (FUSCC).

## 2 MATERIALS AND METHODS

### 2.1 Study population

We obtained data from the current SEER database, which consists of 18 population‐based cancer registries. This database collects and publishes cancer incidence and survival data covering approximately 28% of the total population in the United States. SEER*Stat Version 8.3.4 (http://www.seer.cancer.gov/seerstat) from the National Cancer Institute was used to identify eligible patients in this study.^25^

Because the SEER database began collecting information on the presence or absence of bone metastases at the time of diagnosis in 2010, we included adult patients (≥18 years of age) diagnosed with microscopically confirmed invasive breast cancer between 1 January 2010 and 31 December 2014. We selected patients with only one primary malignancy in their lifetime. In total, 229 195 patients were eligible for inclusion in the incidence analyses. Among whom 8295 were diagnosed with bone metastases. We subsequently removed patients with an unknown follow‐up, leaving 7482 patients eligible for survival analyses.

To validate the preliminary findings obtained from the SEER database, we used data from 198 breast cancer patients with bone metastases at first diagnosis who were treated between January 2004 and December 2017 at FUSCC. All patients included in the analysis were histopathologically reconfirmed independently by two experienced pathologists according to the ASCO/CAP 2010 criteria. The cutoff for estrogen receptor or progesterone receptor positivity was ≥1% of tumor cells with nuclear staining.^26^ Cytoplasmic staining was ignored.^27^ Pathologic HER2 status was defined according to the ASCO/CAP guidelines.^28^

This study was conducted with approval from the Ethical Committee Review Board of Fudan University Shanghai Cancer Center and all patients provided written informed consent.

### 2.2 Statistical analysis

Descriptive statistics were used to examine the baseline characteristics of the patient population. These variables were stratified by breast cancer subtype: HR‐positive HER2‐negative, HR‐positive HER2‐positive, HR‐negative HER2‐positive, and triple‐negative (HR‐negative HER2‐negative). Residence type, median household income, and education level (percentage of adults ≥25 years with a high school education) were estimated with county attributes from the US Census 2010‐2014 American Community Survey 5‐year data files, which were provided through the SEER*Stat software. Patient characteristics were compared among the subgroups with the chi‐square test and Fisher's exact test for categorical variables and with the Kruskal‐Wallis test for continuous variables. Within each variable, patients with unknown data were excluded from the comparative analysis.

Absolute numbers and incidence proportions were calculated for patients with bone metastases at the time of their breast cancer diagnosis. Patients were also stratified by breast cancer subtype. Incidence proportion was defined as the percentage of breast cancer patients diagnosed with bone metastases among either the entire study cohort or the patients with metastatic disease to any distant site.

Multivariable logistic regression was used to determine predictors of the presence of bone metastases at diagnosis. Information regarding the presence of brain, lung, and liver metastases at diagnosis is available in the SEER database and FUSCC cohort and was used to calculate the number of extraosseous metastatic sites in this study.

Overall survival (OS) and breast cancer‐specific survival (BCSS) were the primary study outcomes. OS was defined as the date of diagnosis to the date of death due to any cause or the date of last follow‐up. BCSS was calculated as the time from the date of diagnosis to the date of death attributed to breast cancer or the date of last follow‐up. We used the Kaplan‐Meier method to obtain survival probabilities and analyzed the differences between groups using the log‐rank test. Univariate and multivariate Cox proportional hazard models were applied to assess the independent association of several variables with BCSS and OS, which were reported as hazard ratios and their 95% confidence intervals (95% CIs).

All statistical analyses were performed using R software, version 3.4.3 (http://www.r-project.org) and SPSS software, version 22.0 (SPSS, Chicago, IL, USA). All *P* values reported were two‐sided, and *P* values <0.05 were considered statistically significant.

## 3 RESULTS

### 3.1 Patient characteristics

A total of 229 195 patients in the United States were diagnosed with breast cancer between 2010 and 2014 and were included in the incidence analysis. Table S1 shows the distribution of patient characteristics according to tumor subtype. Most patients had HR‐positive HER2‐negative tumors (66.7%) while the fewest patients had HR‐negative HER2‐positive tumors (4.4%). Within this cohort, 7482 patients had bone metastases at their initial diagnosis and complete survival data; thus, they were included in the survival analysis, and their demographic and clinical characteristics are shown in Table 1. Among the cohort for the survival analysis, 59.5%, 5.9%, 14.9%, 8.2%, and 11.4% of patients had HR‐positive HER2‐negative, HR‐negative HER2‐positive, HR‐positive HER2‐positive, triple‐negative, and unknown subtypes, respectively. At the time of diagnosis, bone was the only site of metastases in 4078 patients (54.5%). Compared with other patients, patients with bone metastases from HR‐positive/HER2‐negative breast cancer were older (*P* < 0.001), were more likely to be white (*P* < 0.001), had a higher rate of lobular histology (*P* < 0.001), had a lower tumor grade (*P* < 0.001), and had a higher household income (*P* < 0.001). In contrast, patients with HR‐negative/HER2‐positive patients were younger (*P* < 0.001), were less likely to be white (*P* < 0.001), had fewer extraosseous metastatic sites to the lung, liver, and brain (*P* < 0.001), and were more likely to live in urban area (*P* = 0.006). The basic characteristics of the patients in the FUSCC cohort are presented in Table S2.

**Table 1:** Demographic characteristics of patients in the SEER cohort included in the survival analysis according to tumor subtypes

Patient characteristics	Tumor subtype										Total		*P*‐value
	HR+/HER2‐		HR‐/HER2+		HR+/HER2+		Triple‐negative		Unknown^a^				
	N	%	N	%	N	%	N	%	N	%	N	%	
All Patients	4454	59.5	445	5.9	1112	14.9	616	8.2	855	11.4	7482	100.0	
Age at diagnose													
18‐49	895	20.1	133	29.9	322	29.0	150	24.4	115	13.5	1615	21.6	<0.001
50‐64	1775	39.9	201	45.2	491	44.2	247	40.1	309	36.1	3023	40.4	
≥65	1784	40.1	111	24.9	299	26.9	219	35.6	431	504	2844	38.0	
Sex													
Female	4400	98.8	443	99.6	1095	98.5	610	99.0	840	98.2	7388	98.7	0.332
Male	54	1.2	2	0.4	17	1.5	6	1.0	15	1.8	94	1.3	
Race													
White	3051	68.5	267	60.0	695	62.5	376	61.0	592	69.2	4981	66.6	<0.001
Black	630	14.1	72	16.2	195	17.5	144	23.4	126	14.7	1167	15.6	
Hispanic	439	9.9	61	13.7	138	12.4	64	10.4	84	9.8	786	10.5	
Asian	292	6.6	40	9.0	75	6.7	26	4.2	44	5.1	477	6.4	
Others^b^	26	0.6	2	0.4	6	0.5	5	0.8	4	0.5	43	0.6	
Unknown^a^	16	0.4	3	0.7	3	0.3	1	0.2	5	0.6	28	0.4	
Laterality													
Left	2154	48.4	221	49.7	573	51.5	307	49.8	375	43.9	3630	48.5	0.486
Right	2145	48.2	215	48.3	512	46.0	285	46.3	322	37.7	3479	46.5	
Bilateral, single primary	21	0.5	5	1.1	7	0.6	3	0.5	16	1.9	52	0.7	
Unknown^a^	134	3.0	4	0.9	20	1.8	21	3.4	142	16.6	321	4.3	
Histology													
IDC	2882	64.7	350	78.7	881	79.2	444	72.1	377	44.1	4934	65.9	<0.001
ILC	703	15.8	16	3.6	45	4.0	31	5.0	1204	6.7	883	11.8	
Others^c^	869	19.5	79	17.8	186	16.7	141	22.9	5041	28.0	1665	22.3	
Grade													
I	406	9.1	3	0.7	21	1.9	12	1.9	36	4.2	478	6.4	<0.001
II	1908	42.8	105	23.6	398	35.8	105	17.0	165	19.3	2681	35.8	
III/IV	1227	27.5	260	58.4	514	46.2	402	65.3	193	22.6	2596	34.7	
Unknown^a^	913	20.5	77	17.3	179	16.1	97	15.7	461	53.9	1727	23.1	
Surgery													
No surgery	3212	72.1	302	67.9	785	70.6	398	64.6	724	84.7	5421	72.5	0.002
BCS	369	8.3	31	7.0	101	9.1	63	10.2	44	5.1	608	8.1	
Mastectomy	844	18.9	108	24.3	217	19.5	149	24.2	74	8.7	1392	18.6	
Unknown^a^	29	0.7	4	0.9	9	0.8	6	1.0	13	1.5	61	0.8	
Extraosseous metastatic sites to lung, liver, and brain, No.													
0	2653	59.6	159	91.5	522	46.9	282	45.8	462	54.0	4078	54.5	<0.001
1	1174	26.4	163	4.9	360	32.4	207	33.6	240	28.1	2144	28.7	
2	339	7.6	76	1.5	150	13.5	77	12.5	77	9.0	719	9.6	
All 3	45	1.0	22	0.3	19	1.7	24	3.9	9	1.1	119	1.6	
Unknown^a^	243	5.5	25	1.9	61	5.5	26	4.2	67	7.8	422	5.6	
Marital status													
Married	1935	43.4	199	44.7	497	44.7	277	45.0	326	38.1	3234	43.2	0.769
Unmarried^d^	2270	51.0	218	49.0	548	49.3	315	51.1	491	57.4	3842	51.3	
Unknown^a^	249	5.6	28	6.3	67	6.0	24	3.9	38	4.4	406	5.4	
Insurance													
Insured	4178	93.8	422	94.8	1024	92.1	576	93.5	799	93.5	6999	93.5	0.272
Uninsured	184	4.1	15	3.4	58	5.2	29	4.7	39	4.6	325	4.3	
Unknown^a^	92	2.1	8	1.8	30	2.7	11	1.8	17	2.0	158	2.1	
Residence type													
Urban	4003	89.9	403	90.6	976	87.8	529	85.9	742	86.8	6653	88.9	0.006
Rural	451	10.1	42	9.4	136	12.2	87	14.1	113	13.2	829	11.1	
Median household income	56 640		56 590		56 490		55 870		56 590		56 590		<0.001
High school education, %	86.7		86.2		85.8		86.1		86.2		86.6		0.083

BCS, breast conserving surgery; HER2, human epidermal growth factor receptor 2; HR, hormone receptor; IDC, infiltrating ductal carcinoma; ILC, infiltrating lobular carcinoma.

a Unknown patients are excluded from the comparative analysis.

b Including American Indian/Alaskan native and Pacific Islander.

c Including other histology of invasive breast cancer except IDC and ILC.

d Including divorced, separated, single (never married), and widowed.

### 3.2 Incidence analysis

**Figure 1:**
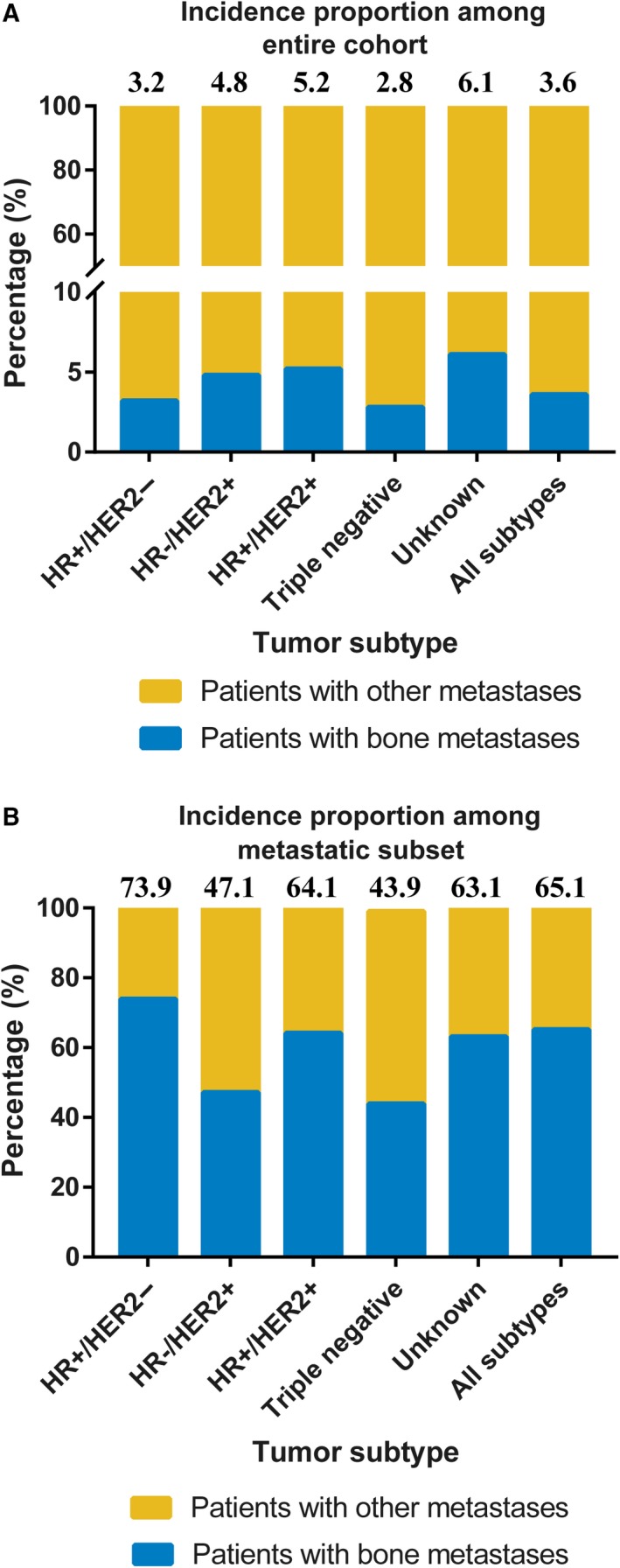
The Incidence Proportion of Patients with Breast Cancer and Bone Metastases at the Time of Initial Diagnosis According to Tumor Subtype in the SEER Cohort. A, The incidence proportion of patients among entire cohort. B, The incidence proportion of patients among subset with metastatic disease. The number above the histogram denotes the incidence proportion of patients with bone metastases. HER2, human epidermal growth factor receptor 2; HR, hormone receptor

Figure 1 shows the number and incidence proportions of patients with breast cancer and bone metastases at diagnosis according to tumor subtype in the SEER cohort. A total of 8295 patients presented with bone metastases, reflecting 3.6% of the entire cohort and 65.1% of the subset with metastatic breast cancer. Patients with the HR‐positive HER2‐negative (3.2% of the entire cohort, 73.9% of the metastatic subset) and HR‐positive HER2‐positive (5.2% of the entire cohort, 64.1% of the metastatic subset) subtypes had the highest incidence proportions.

Multivariable logistic regression was performed among the entire cohort and the subset with metastatic disease in the SEER cohort (Table 2). Among the entire cohort, age 50‐64 years (*P* = 0.003), male sex (*P* = 0.003), infiltrating lobular carcinoma (*P* < 0.001) and other histology (*P* < 0.001), grade II (*P* < 0.001) and III/IV (*P* < 0.001), metastatic disease to 1 extraosseous site (*P* < 0.001), 2 extraosseous sites (*P* < 0.001) and 3 extraosseous sites (*P* < 0.001), uninsured status (*P* < 0.001), unmarried status (*P* < 0.001), and higher education level (*P* < 0.001) were associated with significantly increased risk of bone metastases at diagnosis. Hispanic (*P* = 0.001) and Asian (*P* < 0.001) race, HR‐negative HER2‐positive (*P* < 0.001) and triple‐negative subtypes (*P* < 0.001), higher median household income (*P* < 0.001) were associated with significantly reduced risk of bone metastases at diagnosis. Residence type was not associated with a risk of bone metastases at diagnosis in the multivariable model. Among patients with metastatic cancer, age, race, histology, grade, tumor subtype, extraosseous metastatic sites, and education level were identified as predictors of the presence of bone metastases at diagnosis.

**Table 2:** Multivariable logistic regression for the presence of bone metastases at diagnosis of breast cancer in the SEER cohort

Patient characteristics	Among entire cohort		Among subset with metastatic disease	
	OR (95% CI)	*P*‐value	OR (95% CI)	*P*‐value
Age at diagnosis				
18‐49	Reference	Reference	Reference	Reference
50‐64	1.109 (1.036‐1.188)	0.003	1.000 (0.899‐1.112)	0.996
≥65	1.000 (0.931‐1.073)	0.993	0.826 (0.740‐0.922)	0.001
Sex				
Female	Reference	Reference	Reference	Reference
Male	1.450 (1.136‐1.850)	0.003	1.207 (0.835‐1.744)	0.317
Race				
White	Reference	Reference	Reference	Reference
Black	0.989 (0.915‐1.068)	0.773	0.808 (0.725‐0.901)	<0.001
Hispanic	0.864 (0.792‐0.942)	0.001	0.893 (0.783‐1.019)	0.093
Asian	0.775 (0.699‐0.859)	<0.001	0.874 (0.747‐1.021)	0.090
Others^a^	0.798 (0.564‐1.130)	0.203	0.897 (0.549‐1.466)	0.666
Histology				
IDC	Reference	Reference	Reference	Reference
ILC	1.996 (1.840‐2.165)	<0.001	1.243 (1.065‐1.451)	0.006
Others^b^	1.238 (1.157‐1.325)	<0.001	0.927 (0.837‐1.026)	0.143
Grade				
I	Reference	Reference	Reference	Reference
II	2.449 (2.219‐2.702)	<0.001	1.125 (0.928‐1.365)	0.229
III/IV	2.872 (2.587‐3.187)	<0.001	0.712 (0.587‐0.863)	0.001
Unknown	7.576 (6.770‐8.478)	<0.001	0.992 (0.813‐1.212)	0.940
Tumor subtype				
HR+/HER‐	Reference	Reference	Reference	Reference
HR‐/HER2+	0.618 (0.548‐0.698)	<0.001	0.390 (0.338‐0.450)	<0.001
HR+/HER2+	1.063 (0.981‐1.151)	0.136	0.737 (0.656‐0.828)	<0.001
Triple‐negative	0.470 (0.426‐0.519)	<0.001	0.340 (0.301‐0.384)	<0.001
Unknown	0.780 (0.624‐0.788)	<0.001	0.669 (0.591‐0.756)	<0.001
Extraosseous metastatic sites, No.				
0	Reference	Reference	Reference	Reference
1	44.896 (41.868‐48.143)	<0.001	0.391 (0.358‐0.426)	<0.001
2	89.690 (78.928‐101.919)	<0.001	0.814 (0.709‐0.934)	0.003
All 3	192.634 (130.771‐283.765)	<0.001	1.895 (1.286‐2.792)	0.001
Unknown	4.906 (4.396‐5.475)	<0.001	0.494 (0.423‐0.577)	<0.001
Marital status				
Married	Reference	Reference	Reference	Reference
Unmarried^c^	1.367 (1.296‐1.443)	<0.001	1.049 (0.966‐1.139)	0.258
Unknown	0.959 (0.852‐1.079)	0.483	0.894 (0.755‐1.060)	0.197
Insurance				
Insured	Reference	Reference	Reference	Reference
Uninsured	1.592 (1.387‐1.827)	<0.001	1.069 (0.887‐1.289)	0.483
Unknown	0.571 (0.476‐0.683)	<0.001	0.743 (0.577‐0.955)	0.021
Residence type				
Urban	Reference	Reference	Reference	Reference
Rural	0.992 (0.906‐1.086)	0.863	0.908 (0.792‐1.042)	0.169
Median household income (per $10 000 annual increase)	0.951 (0.931‐0.972)	<0.001	0.977 (0.946‐1.010)	0.173
High school education (per 10% increase)	1.066 (1.024‐1.111)	0.002	1.081 (1.016‐1.151)	0.015

CI, confidence interval; HER2, human epidermal growth factor receptor 2; HR, hormone receptor; IDC, infiltrating ductal carcinoma; ILC, infiltrating lobular carcinoma.

Unknown age and unknown race removed from model owing to nonconvergence (n = 1798).

a Including American Indian/Alaskan native and Pacific Islander.

b Including other histology of invasive breast cancer except IDC and ILC.

c Including divorced, separated, single (never married), and widowed.

### 3.3 Survival analysis

**Figure 2:**
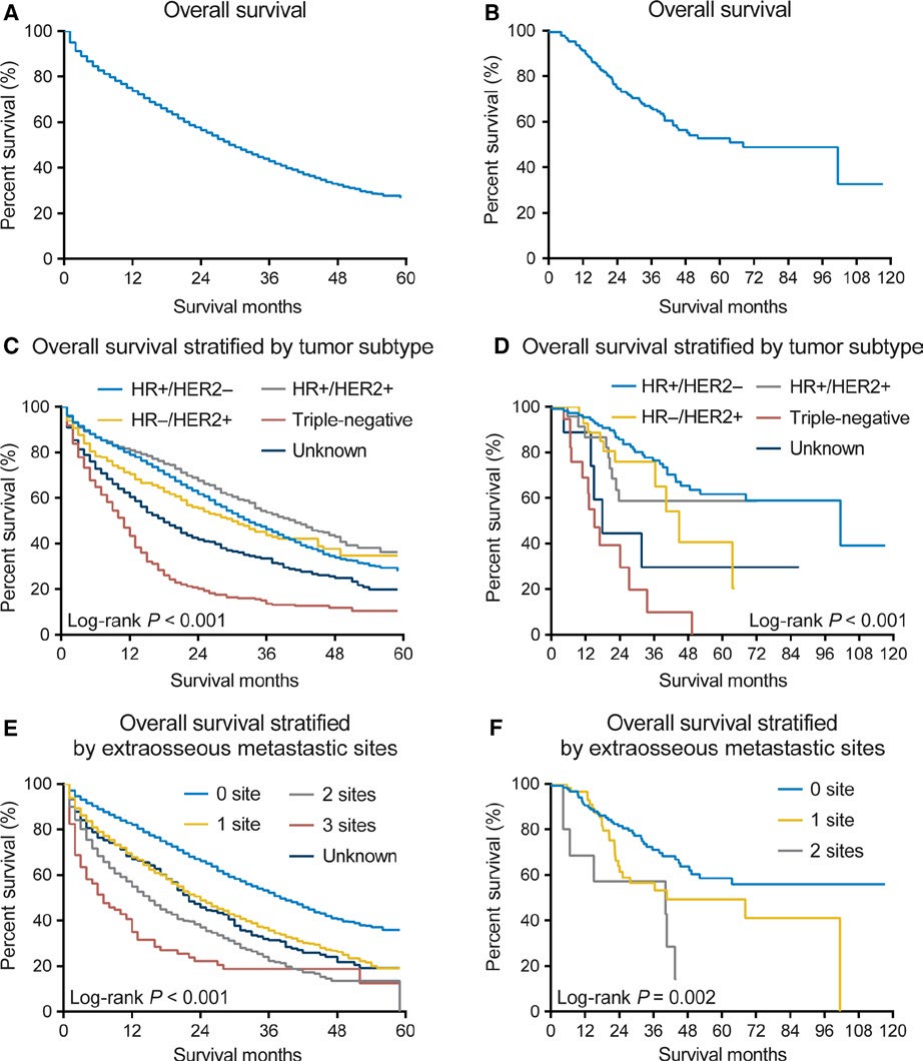
Kaplan‐Meier curves for Overall Survival Among Patients with Breast Cancer and Bone Metastases at the Time of Initial Diagnosis in both SEER and FUSCC Cohorts. A‐B, The whole population included in the survival analysis in the (A) SEER and (B) FUSCC cohort, respectively. C‐D, According to the tumor subtype in the (C) SEER and (D) FUSCC cohort, respectively. E‐F, According to the number of extraosseous metastatic sites to lung, liver, and brain in the (E) SEER and (F) FUSCC cohort, respectively. HER2, human epidermal growth factor receptor 2; HR, hormone receptor

The median OS of the SEER cohort and FUSCC cohort included in the survival analysis was 30 and 68.2 months, respectively (Figure 2A,B). Significant differences were observed from the OS analysis according to tumor subtype (Figure 2C,D; log‐rank *P* < 0.001). Patients with the HR‐positive HER2‐positive subtype experienced the longest median survival in both SEER cohort and FUSCC cohort (41.0 months and not reached, respectively), whereas patients with the triple‐negative subtype experienced the shortest median survival (11.0 and 15.1 months, respectively). Survival estimates stratified by extraosseous metastatic sites are displayed in Figure 2E,F; and patients with more extraosseous metastatic sites showed worse OS (log‐rank *P* < 0.001 in the SEER cohort and log‐rank *P* = 0.002 in the FUSCC cohort).

**Figure 3:**
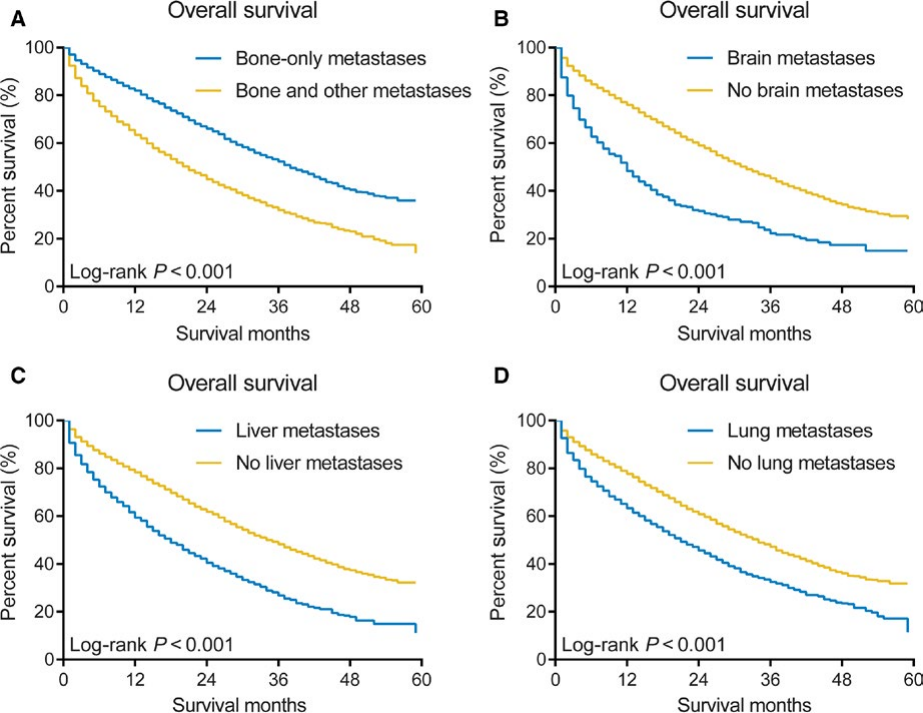
Kaplan‐Meier curves for Overall Survival According to Individual Metastases in the SEER Cohort. A, Patients with bone‐only metastases vs those with bone and other metastases. B, Patients with brain metastases vs those without brain metastases. C, Patients with liver metastases vs those without liver metastases. D, Patients with lung metastases vs those without lung metastases

The impact of the presence of extraosseous metastases on OS in the SEER cohort is shown in Figure 3. Patients with metastases to the bone and other sites had significantly shorter survival (median OS: 21.0 months) than those with metastases to the bone only (median OS: 38.0 months, log‐rank *P* < 0.001). There were also significant differences in OS between patients with liver metastases vs those without liver metastases (median OS: 18.0 vs 35.0 months, log‐rank *P* < 0.001), patients with lung metastases vs those without lung metastases (median OS: 21.0 vs 34.0 months, log‐rank *P* < 0.001), and patients with brain metastases vs those without brain metastases (median OS: 12.0 vs 32.0 months, log‐rank *P* < 0.001).

We used univariate and multivariate Cox proportional hazard models to determine the prognostic factors of breast cancer patients with bone metastases at diagnosis in the SEER cohort, the results of which are shown in Table 3. In the univariate analysis, age, tumor grade, tumor subtype, extraosseous metastatic sites, history of surgery, insurance status, marital status, residence type, median household income, and high school education level were significantly associated with OS and BCSS (*P* < 0.05). Multivariable Cox analysis confirmed that age, histology, tumor grade, tumor subtype, extraosseous metastatic sites, history of primary surgery, insurance status, marital status, and median household income are independent prognostic factors for both OS and BCSS (*P* < 0.05). Sex, race, residence type, and education level did not reach significance in the multivariable test. We also found that there is no significant difference of survival time between patients with de novo osseous and extraosseous metastatic disease, except for patients with liver metastases at initial diagnosis (Table S3).

**Table 3:** Cox regression analysis of overall survival and breast cancer‐specific survival among patients with bone metastases in the SEER cohort

Patient characteristics	Overall survival				Breast cancer‐specific survival			
	Univariate analysis		Multivariate analysis		Univariate analysis		Multivariate analysis	
	Hazard ratio (95% CI)	*P*‐value	Hazard ratio (95% CI)	*P*‐value	Hazard ratio (95% CI)	*P*‐value	Hazard ratio (95% CI)	*P*‐value
Age at diagnosis								
18‐49	Reference		Reference		Reference		Reference	
50‐64	1.398 (1.268‐1.542)	<0.001	1.334 (1.208‐1.472)	<0.001	1.398 (1.262‐1.547)	<0.001	1.331 (1.201‐1.475)	<0.001
≥65	1.861 (1.690‐2.049)	<0.001	1.782 (1.611‐1.970)	<0.001	1.776 (1.606‐1.965)	<0.001	1.702 (1.532‐1.891)	<0.001
Sex								
Female	Reference		Reference		Reference			
Male	1.061 (0.783‐1.439)	0.702	1.063 (0.782‐1.444)	0.697	0.970 (0.695‐1.354)	0.858	0.979 (0.700‐1.369)	0.901
Race								
White	Reference		Reference		Reference		Reference	
Black	1.258 (1.148‐1.378)	<0.001	1.095 (0.995‐1.204)	0.062	1.233 (1.120‐1.357)	<0.001	1.061 (0.960‐1.173)	0.243
Hispanic	0.902 (0.804‐1.012)	0.079	0.961 (0.853‐1.083)	0.512	0.889 (0.788‐1.003)	0.056	0.938 (0.827‐1.063)	0.314
Asian	0.900 (0.778‐1.041)	0.154	0.976 (0.842‐1.132)	0.747	0.883 (0.758‐1.030)	0.114	0.954 (0.816‐1.115)	0.555
Others^a^	0.893 (0.554‐1.439)	0.641	0.962 (0.595‐1.555)	0.875	0.977 (0.606‐1.575)	0.924	1.049 (0.648‐1.696)	0.846
Histology								
IDC	Reference		Reference		Reference		Reference	
ILC	1.007 (0.904‐1.121)	0.900	1.189 (1.061‐1.333)	0.003	1.008 (0.900‐1.128)	0.892	1.213 (1.077‐1.368)	0.002
Others^b^	1.291 (1.193‐1.398)	<0.001	1.161 (1.063‐1.269)	0.001	1.284 (1.182‐1.396)	<0.001	1.152 (1.049‐1.264)	0.003
Grade								
I	Reference		Reference		Reference		Reference	
II	1.279 (1.085‐1.507)	0.003	1.293 (1.095‐1.527)	0.002	1.344 (1.126‐1.604)	0.001	1.360 (1.137‐1.626)	0.001
III/IV	1.861 (1.582‐2.189)	<0.001	1.892 (1.598‐2.241)	<0.001	1.984 (1.666‐2.362)	<0.001	1.998 (1.666‐2.395)	<0.001
Tumor subtype								
HR+/HER‐	Reference		Reference		Reference		Reference	
HR‐/HER2+	1.165 (1.003‐1.353)	0.045	1.022 (0.876‐1.191)	0.786	1.220 (1.046‐1.423)	0.012	1.049 (0.895‐1.230)	0.553
HR+/HER2+	0.829 (0.743‐0.925)	0.001	0.749 (0.669‐0.838)	<0.001	0.835 (0.744‐0.937)	0.002	0.747 (0.664‐0.841)	<0.001
Triple‐negative	2.868 (2.582‐3.185)	<0.001	2.530 (2.265‐2.826)	<0.001	2.959 (2.652‐3.301)	<0.001	2.584 (2.303‐2.900)	<0.001
Unknown	1.673 (1.515‐1.848)	<0.001	1.407 (1.268‐1.560)	<0.001	1.718 (1.549‐1.905)	<0.001	1.450 (1.302‐1.616)	<0.001
Extraosseous metastatic sites, No.								
0	Reference		Reference		Reference		Reference	
1	1.662 (1.538‐1.797)	<0.001	1.546 (1.428‐1.675)	<0.001	1.705 (1.571‐1.850)	<0.001	1.589 (1.461‐1.727)	<0.001
2	2.457 (2.210‐2.732)	<0.001	2.275 (2.040‐2.538)	<0.001	2.556 (2.289‐2.854)	<0.001	2.365 (2.111‐2.650)	<0.001
All 3	3.674 (2.948‐4.579)	<0.001	3.184 (2.544‐3.984)	<0.001	3.960 (3.163‐4.958)	<0.001	3.404 (2.707‐4.281)	<0.001
Surgery								
No	Reference		Reference		Reference		Reference	
BCS	0.485 (0.420‐0.559)	<0.001	0.527 (0.455‐0.609)	<0.001	0.495 (0.427‐0.574)	<0.001	0.542 (0.465‐0.631)	<0.001
Mastectomy	0.528 (0.480‐0.581)	<0.001	0.565 (0.511‐0.625)	<0.001	0.531 (0.480‐0.587)	<0.001	0.569 (0.512‐0.633)	<0.001
Insurance								
Insured	Reference		Reference		Reference		Reference	
Uninsured	1.252 (1.068‐1.466)	0.006	1.186 (1.009‐1.395)	0.039	1.263 (1.071‐1.490)	0.006	1.186 (1.002‐1.405)	0.048
Marital status								
Married			Reference				Reference	
Unmarried^c^	1.425 (1.327‐1.529)	<0.001	1.304 (1.213‐1.402)	<0.001	1.400 (1.300‐1.508)	<0.001	1.290 (1.196‐1.392)	<0.001
Residence type								
Urban	Reference		Reference		Reference		Reference	
Rural	1.118 (1.007‐1.241)	0.036	1.015 (0.902‐1.141)	0.808	1.119 (1.003‐1.248)	0.044	0.984 (0.870‐1.113)	0.800
Median household income (per $10 000 annual increase)	0.940 (0.919‐0.962)	<0.001	0.948 (0.920‐0.976)	<0.001	0.933 (0.910‐0.955)	<0.001	0.937 (0.909‐0.966)	<0.001
High school education (per 10% increase)	0.931 (0.889‐0.975)	0.002	0.968 (0.917‐1.022)	0.241	0.925 (0.882‐0.970)	0.001	0.969 (0.916‐1.025)	0.271

CI, confidence interval; HER2, human epidermal growth factor receptor 2; HR, hormone receptor IDC, infiltrating ductal carcinoma; ILC, infiltrating lobular carcinoma.

We hid the unknown data in order to avoid confusion.

a Including American Indian/Alaskan native and Pacific Islander.

b Including other histology of invasive breast cancer except IDC and ILC.

c Including divorced, separated, single (never married), and widowed.

## 4 DISCUSSION

This study analyzed recently available data from the SEER database on the incidence proportion and survival of patients with metastatic breast cancer who had bone metastases at their initial diagnosis. We also validated the results of survival analysis in another independent cohort from FUSCC. Some studies have evaluated the epidemiology and prognosis of breast cancer patients with bone metastases at the population level, but most of the patients in these studies presented bone metastases after a diagnosis of early‐stage breast cancer.^2^, ^20^, ^29^, ^30^, ^31^, ^32^, ^33^ However, limited data have been reported in the specific group of patients with bone metastases upon their initial diagnosis of breast cancer.^34^ Because early detection and systemic treatment of bone metastases in patients with breast cancer may modify the natural progression of bone metastases, reduce the probability of SREs, and improve progression‐free survival, quality of life and cost‐effective care, it is important for us to study patients who present with de novo bone metastases in a large independent cohort.

In this large retrospective study, we found that 3.6% of patients with invasive breast cancer had bone metastases at diagnosis, and 65.1% of those with any metastases at diagnosis had bone metastases. This result is slightly different from that of a previously published meta‐analysis.^35^ Body et al included and analyzed observational studies and clinical trials published between 1999 and 2013. The median (range) proportion of patients who had bone metastases was 14.7% (1.4%‐61.8%) in the breast cancer cohort and 58.3% (18.2%‐91.7%) in the metastatic cohort. This difference is possibly since consensus guidelines for patients with early‐stage breast cancer do not recommend a bone scan unless signs or symptoms have developed. As a result, the incidence proportion of bone metastases in patients with breast cancer in our study is likely underestimated.

We also observed significant differences in demographic and clinical characteristics of patients according to tumor subtype. Some of the relative high‐risk features in patients with triple‐negative breast cancer included black race, higher tumor grade, more extraosseous metastatic sites, and lower rate of primary tumor surgery. In addition, our study demonstrated that different tumor subtypes showed a different tendency to bone metastases. The incidence proportion of bone metastases was highest among patients with the HR‐positive HER2‐negative and HR‐positive HER2‐positive subtypes, which are in accordance with other publications describing the patterns of metastatic breast cancer.^11^, ^12^, ^36^ The multivariable logistic regression indicated that patients with the HR‐positive HER2‐negative subtype had significantly greater odds of having bone metastases at diagnosis than patients with other subtypes, whether among the entire cohort or within the subset with metastatic disease. Previous findings have confirmed that patients with an HR‐positive status are more likely to have bone metastases than those with HR‐negative status. The absence of WNT/β‐catenin signaling and the involvement of transforming growth factor β and fibroblast growth factor signaling have been found to promote HR‐positive breast cancer metastases to bone.^12^, ^37^

We identified predictors of the presence of bone metastases at diagnosis with the use of multivariate logistic regression to distinguish patients at increased risk of bone metastases. Our results could serve as a basis for future studies to evaluate the utility of a bone scan among these high‐risk patients. Among the entire cohort, we found that patients who were uninsured (vs insured, OR, 1.592; 95% CI, 1.387‐1.827; *P* < 0.001), were unmarried (vs married, OR, 1.367; 95% CI, 1.296‐1.443; *P* < 0.001) and had a higher school education (per 10% increase, OR, 1.066; 95% CI, 1.024‐1.111; *P* = 0.002) had a significantly greater likelihood of presenting bone metastases at diagnosis, but these associations were not observed among the cohort with metastatic breast cancer at diagnosis (except for the education level). Patients with uninsured status and unmarried status are more likely to ignore symptoms or signs and be diagnosed at late stage. However, there is no research studying this association and further study for this finding is warranted.

The median OS was 30 and 68.2 months from initial diagnosis in the SEER cohort and FUSCC cohort, respectively, which is similar with the survival reported by previous authors.^20^, ^29^, ^30^, ^33^ We assume that the longer survival in the FUSCC cohort was because the patients in this cohort were much younger and had less extraosseous metastatic sites. Notably, we found that the median survival for patients with breast cancer and bone metastases varied significantly by tumor subtypes. Compared with HR‐positive HER2‐negative patients, HR‐positive HER2‐positive patients experienced a 25.1% reduction in the hazards of overall mortality while triple‐negative patients experienced a 153.0% increase in the hazards of overall mortality. Our data are consistent with some previous retrospective studies.^22^, ^23^, ^38^, ^39^ We found that HR status in patients who are HER2‐positive is an important prognostic factor. Patients with bone metastases could be treated with chemotherapy, endocrine therapy or HER2‐targeted therapy according to the tumor subtypes. HR‐positive HER2‐positive have the option to undergo endocrine therapy, which may improve OS; HR‐negative HER2‐positive patients do not have this option.^40^

The presence of extraosseous disease in patients with bone metastases had a worse impact on survival. A recent study showed that compared with patients with visceral metastases with or without bone metastases, patients with bone‐only metastases at start of first‐line therapy had an improved median OS (54 months vs 28 months).^29^ Other studies also found that the median survival of patients with bone‐only metastases was approximately twofold to threefold that of patients with additional visceral metastases.^30^, ^41^, ^42^, ^43^ The results of our cohort study reached the same conclusion that patients with metastases to the bone only had better survival than those with metastases to both the bone and extraosseous sites. In addition, we found that patients with more extraosseous sites had worse survival, even after adjusting for other prognostic factors in the multivariate model. When we analyzed specific extraosseous metastatic sites by the log‐rank test, we identified that the presence of metastases at another sites such as the brain, liver, or lung had a significant negative impact on OS. We speculate that the improved survival in patients with bone‐only metastases patients is because bone is not a vital organ, and patients with bone‐only metastases have a slower onset of vital organ dysfunction. In general, our findings underscore that tumor subtypes and properties may be associated with the correlation between the clinical aggressiveness of the tumor and the metastases to specific sites.

Some limitations of our study should be acknowledged. First, information relating to recurrence or metastases after a diagnosis of breast cancer is not available in the SEER database. Therefore, we were unable to obtain and analyze the data on patients who showed progression in their disease course. Second, for patients with early‐stage breast cancer, routine screening for bone metastases is recommended only if directed by signs or symptoms. However, a subset of patients with bone metastases do not present symptoms, resulting in an underestimation of the actual rate of patients with de novo bone metastases. Third, we do not have information about systemic treatment, such as endocrine therapy, HER2‐targeted therapy, or chemotherapy, which may contribute to some bias in the survival analysis. Fourth, the SEER database only provides information about four sites of metastases at diagnosis: bone, brain, lung, and liver. Information on other sites of metastases such as the pleura and skin is lacking, which may influence the prognostic assessment of the extraosseous metastases group. Fifth, the finding of our analysis is limited to the United States, as some socioeconomic factors such as insurance, income, and education level are different in other parts of the world. Finally, residence type, median household income, and education level were defined at the county level, not the patient level. This may cause deviations in the analysis.

To the best our knowledge, our study is the first population‐based analysis of patients with bone metastases upon initial diagnosis of breast cancer. It provides important information for clinicians to consider conducting studies that evaluate the utility of a bone scan among patients at higher risk for bone metastases. Our study does not suffer from confounding effects that systemic therapies might cause on the timing of development and potential drug resistance of bone metastases, so the findings about the prognostic impact of tumor subtypes and extraosseous metastatic sites could be used for prognostic assessments and risk stratification of breast cancer patients with bone metastases at initial diagnosis. However, whether an earlier diagnosis of bone metastases may impact outcomes warrants further investigation.

## ETHICS APPROVAL AND CONSENT TO PARTICIPATE

Our study was approved by Shanghai Cancer Center Ethical Committee. Cancer a reportable disease in every state in the United States; informed patient consent is not required for the data released by the SEER database.

## AVAILABILITY OF DATA AND MATERIAL

The datasets generated and analyzed during this study are available from Surveillance, Epidemiology, and End Results (SEER) Program (http://www.seer.cancer.gov) SEER*Stat Database: Incidence—SEER 18 Regs Research Data + Hurricane Katrina Impacted Louisiana Cases, Nov 2016 Sub (1973‐2014 varying), National Cancer Institute, DCCPS, Surveillance Research Program, released April 2017, based on the November 2016 submission.

## CONFLICT OF INTEREST

The authors declare no conflict of interest.
