# Supplementary material for: Incidence proportions and prognosis of breast cancer patients with bone metastases at initial diagnosis
Source: Cancer Med. 2018 Jul 9;7(8):4156–69. doi: 10.1002/cam4.1668 (PMC6089179; doi:10.1002/cam4.1668)
Supplement: Supplementary file 1 [file CAM4-7-4156-s001.docx]

| **Supplementary Table 1.** Demographic characteristics of patients among entire cohort according to tumor subtypes | | | | | | | | | | | | | | | | | | |
| --- | --- | --- | --- | --- | --- | --- | --- | --- | --- | --- | --- | --- | --- | --- | --- | --- | --- | --- |
| **Patient characteristics** | **Tumor subtype** | | | | | | | | | | | | | |  | **Total** | | ***P*-Value** |
|  | **HR+/HER2-** | |  | **HR-/HER2+** | |  | **HR+/HER2+** | |  | **Triple negative** | |  | **Unknown**† | |  |  |  |  |
|  | **N** | **%** |  | **N** | **%** |  | **N** | **%** |  | **N** | **%** |  | **N** | **%** |  | **N** | **%** |  |
| All Patients | 152,977 | 66.7 |  | 10,154 | 4.4 |  | 23,218 | 10.1 |  | 24,834 | 10.8 |  | 18,102 | 7.9 |  | 229,195 | 100.0 |  |
| Age at diagnose, y |  |  |  |  |  |  |  |  |  |  |  |  |  |  |  |  |  | <0.001 |
| 18-49 | 31,583 | 20.6 |  | 2,903 | 28.6 |  | 7,226 | 31.1 |  | 7,313 | 29.4 |  | 3,750 | 20.8 |  | 52,775 | 23.0 |  |
| 50-64 | 58,537 | 38.3 |  | 4,514 | 44.5 |  | 9,576 | 41.2 |  | 9,809 | 39.5 |  | 6,628 | 36.8 |  | 89,064 | 38.9 |  |
| ≥65 | 62,857 | 41.1 |  | 2,737 | 27.0 |  | 6,416 | 27.6 |  | 7,712 | 31.1 |  | 7,634 | 42.4 |  | 87,356 | 38.1 |  |
| Sex |  |  |  |  |  |  |  |  |  |  |  |  |  |  |  |  |  | <0.001 |
| Female | 151,687 | 99.2 |  | 10,140 | 99.9 |  | 23,042 | 99.2 |  | 24,804 | 99.9 |  | 17,816 | 98.9 |  | 227,489 | 99.3 |  |
| Male | 1,290 | 0.8 |  | 14 | 0.1 |  | 176 | 0.8 |  | 30 | 0.1 |  | 196 | 1.1 |  | 1,706 | 0.7 |  |
| Race |  |  |  |  |  |  |  |  |  |  |  |  |  |  |  |  |  | <0.001 |
| White | 107,128 | 70.0 |  | 5,898 | 58.1 |  | 14,762 | 63.6 |  | 14,680 | 59.1 |  | 11,280 | 62.6 |  | 153,748 | 67.1 |  |
| Black | 14,296 | 9.3 |  | 1,409 | 13.9 |  | 2,792 | 12.0 |  | 5,047 | 20.3 |  | 2,156 | 12.0 |  | 25,700 | 11.2 |  |
| Hispanic | 16,684 | 10.9 |  | 1,422 | 14.0 |  | 3,040 | 13.1 |  | 3,127 | 12.6 |  | 2,462 | 13.7 |  | 26,735 | 11.7 |  |
| Asian | 13,018 | 8.5 |  | 1,293 | 12.7 |  | 2,300 | 9.9 |  | 1,720 | 6.9 |  | 1,568 | 8.7 |  | 19,899 | 8.7 |  |
| Others‡ | 842 | 0.6 |  | 68 | 0.7 |  | 168 | 0.7 |  | 141 | 0.6 |  | 96 | 0.5 |  | 1,315 | 0.6 |  |
| Unknown† | 1,009 | 0.7 |  | 64 | 0.6 |  | 156 | 0.7 |  | 119 | 0.5 |  | 450 | 2.5 |  | 1,798 | 0.8 |  |
| Laterality |  |  |  |  |  |  |  |  |  |  |  |  |  |  |  |  |  | <0.001 |
| Left | 77,105 | 50.4 |  | 5,248 | 51.7 |  | 11,883 | 51.2 |  | 12,721 | 51.2 |  | 8,964 | 49.8 |  | 115,921 | 50.6 |  |
| Right | 75,559 | 49.4 |  | 4,877 | 48.0 |  | 11,279 | 48.6 |  | 12,049 | 48.5 |  | 8,344 | 46.3 |  | 112,108 | 48.9 |  |
| Bilateral, single primary | 48 | 0.0 |  | 12 | 0.1 |  | 12 | 0.1 |  | 7 | 0.0 |  | 34 | 0.2 |  | 113 | 0.0 |  |
| Unknown† | 265 | 0.2 |  | 17 | 0.2 |  | 44 | 0.2 |  | 57 | 0.2 |  | 670 | 3.7 |  | 1,053 | 0.5 |  |
| Histology |  |  |  |  |  |  |  |  |  |  |  |  |  |  |  |  |  | <0.001 |
| IDC | 110,631 | 72.3 |  | 8,920 | 87.8 |  | 19,748 | 85.1 |  | 20,979 | 84.5 |  | 11,767 | 65.3 |  | 172,045 | 75.1 |  |
| ILC | 17,029 | 11.1 |  | 85 | 0.8 |  | 809 | 3.5 |  | 279 | 1.1 |  | 1,204 | 6.7 |  | 19,406 | 8.5 |  |
| Others§ | 25,317 | 16.5 |  | 1,149 | 11.3 |  | 2,661 | 11.5 |  | 3,576 | 14.4 |  | 5,041 | 28.0 |  | 37,744 | 16.5 |  |
| Grade |  |  |  |  |  |  |  |  |  |  |  |  |  |  |  |  |  | <0.001 |
| I | 42,922 | 28.1 |  | 138 | 1.4 |  | 1,380 | 5.9 |  | 485 | 2.0 |  | 2,764 | 15.3 |  | 47,689 | 20.8 |  |
| II | 72,473 | 47.4 |  | 2,196 | 21.6 |  | 8,918 | 38.4 |  | 3,960 | 15.9 |  | 5,587 | 31.0 |  | 93,134 | 40.6 |  |
| III/IV | 31,039 | 20.3 |  | 7,021 | 69.1 |  | 11,607 | 50.0 |  | 19,115 | 77.0 |  | 4,783 | 26.6 |  | 73,565 | 32.1 |  |
| Unknown† | 6,543 | 4.3 |  | 799 | 7.9 |  | 1,313 | 5.7 |  | 1,274 | 5.1 |  | 4,878 | 27.1 |  | 14,807 | 6.5 |  |
| Surgery |  |  |  |  |  |  |  |  |  |  |  |  |  |  |  |  |  | <0.001 |
| No surgery | 10,753 | 7.0 |  | 1,381 | 13.6 |  | 2,658 | 11.4 |  | 2,410 | 9.7 |  | 4,043 | 22.4 |  | 21,245 | 9.3 |  |
| BCS | 87,008 | 56.9 |  | 3,594 | 35.4 |  | 9,913 | 42.7 |  | 11,610 | 46.8 |  | 7,376 | 41.0 |  | 119,501 | 52.1 |  |
| Mastectomy | 54,836 | 35.8 |  | 5,131 | 50.5 |  | 10,560 | 45.5 |  | 10,745 | 43.3 |  | 6,254 | 34.7 |  | 87,526 | 38.2 |  |
| Unknown† | 380 | 0.2 |  | 48 | 0.5 |  | 87 | 0.4 |  | 69 | 0.3 |  | 339 | 1.9 |  | 923 | 0.4 |  |
| Extraosseous metastatic sites to lung, liver and brain, No. |  |  |  |  |  |  |  |  |  |  |  |  |  |  |  |  |  | <0.001 |
| 0 | 148,510 | 97.1 |  | 9,286 | 91.5 |  | 21,797 | 93.9 |  | 23,571 | 94.9 |  | 15,487 | 86.0 |  | 218,651 | 95.4 |  |
| 1 | 2,087 | 1.4 |  | 494 | 4.9 |  | 782 | 3.4 |  | 693 | 2.8 |  | 589 | 3.3 |  | 4,645 | 2.0 |  |
| 2 | 504 | 0.3 |  | 150 | 1.5 |  | 225 | 1.0 |  | 201 | 0.8 |  | 194 | 1.1 |  | 1,274 | 0.6 |  |
| All 3 | 64 | 0.0 |  | 32 | 0.3 |  | 26 | 0.1 |  | 35 | 0.1 |  | 26 | 0.1 |  | 183 | 0.1 |  |
| Unknown† | 1,812 | 1.2 |  | 192 | 1.9 |  | 388 | 1.7 |  | 334 | 1.3 |  | 1,716 | 9.5 |  | 4,442 | 1.9 |  |
| Marital status |  |  |  |  |  |  |  |  |  |  |  |  |  |  |  |  |  | <0.001 |
| Married | 84,404 | 55.2 |  | 5,584 | 55.0 |  | 13,057 | 56.2 |  | 13,142 | 52.9 |  | 8,380 | 46.5 |  | 124,567 | 54.3 |  |
| Unmarried¶ | 60,126 | 39.3 |  | 4,004 | 39.4 |  | 8,931 | 38.5 |  | 10,306 | 41.5 |  | 7,552 | 41.9 |  | 90,919 | 39.7 |  |
| Unknown† | 8,447 | 5.5 |  | 566 | 5.6 |  | 1,230 | 5.3 |  | 1,386 | 5.6 |  | 2,080 | 11.5 |  | 13,709 | 6.0 |  |
| Insurance |  |  |  |  |  |  |  |  |  |  |  |  |  |  |  |  |  | <0.001 |
| Insured | 147,511 | 96.4 |  | 9,712 | 95.6 |  | 22,203 | 95.6 |  | 23,761 | 95.7 |  | 16,024 | 89.0 |  | 219,211 | 95.6 |  |
| Uninsured | 2,523 | 1.6 |  | 261 | 2.6 |  | 572 | 2.5 |  | 653 | 2.6 |  | 496 | 2.8 |  | 4,505 | 2.0 |  |
| Unknown† | 2,943 | 1.9 |  | 181 | 1.8 |  | 443 | 1.9 |  | 420 | 1.7 |  | 1,492 | 8.3 |  | 5,479 | 2.4 |  |
| Residence type |  |  |  |  |  |  |  |  |  |  |  |  |  |  |  |  |  | <0.001 |
| Urban | 137,988 | 90.2 |  | 9,146 | 90.1 |  | 20,945 | 90.2 |  | 22,169 | 89.3 |  | 15,903 | 88.3 |  | 206,151 | 89.9 |  |
| Rural | 14,989 | 9.8 |  | 1,008 | 9.9 |  | 2,273 | 9.8 |  | 2,665 | 10.7 |  | 2,109 | 11.7 |  | 23,044 | 10.1 |  |
| Median household income | 58,970 |  |  | 56,590 |  |  | 56,640 |  |  | 55,870 |  |  | 56,590 |  |  | 56,640 |  | <0.001 |
| High-school education, % | 86.7 |  |  | 86.6 |  |  | 86.3 |  |  | 86.2 |  |  | 85.7 |  |  | 86.7 |  | <0.001 |
| Abbreviations: BCS, breast conserving surgery; IDC, infiltrating ductal carcinoma; ILC, infiltrating lobular carcinoma; HER2, human epidermal growth factor receptor 2; HR, hormone receptor.  † Unknown patients are excluded from the comparative analysis.  ‡ Including American Indian/Alaskan native and Pacific Islander.  § Including other histology of invasive breast cancer except IDC and ILC.  ¶ Including divorced, separated, single (never married), and widowed. | | | | | | | | | | | | | | | | | | |
