# Supplementary material for: Incidence proportions and prognosis of breast cancer patients with bone metastases at initial diagnosis
Source: Cancer Med. 2018 Jul 9;7(8):4156–69. doi: 10.1002/cam4.1668 (PMC6089179; doi:10.1002/cam4.1668)
Supplement: Supplementary file 2 [file CAM4-7-4156-s002.docx]

| **Supplementary Table 2.** Demographic characteristics of patients in the FUSCC cohort included in the survival analysis | | |
| --- | --- | --- |
| **Patient characteristics** | **No. of patients (N = 198)** | **%** |
| Age at diagnose, y |  |  |
| 18-49 | 101 | 51.0 |
| 50-64 | 71 | 35.9 |
| ≥65 | 26 | 13.1 |
| Sex |  |  |
| Female | 196 | 99.0 |
| Male | 2 | 1.0 |
| Laterality |  |  |
| Left | 92 | 46.5 |
| Right | 93 | 47.0 |
| Bilateral, single primary | 13 | 6.5 |
| Histology |  |  |
| IDC | 157 | 79.3 |
| ILC | 21 | 10.6 |
| Others† | 20 | 10.1 |
| Grade |  |  |
| I | 9 | 4.5 |
| II | 86 | 43.4 |
| III | 92 | 46.5 |
| Unknown | 11 | 5.6 |
| Subtype |  |  |
| HR+/HER2- | 112 | 56.6 |
| HR-/HER2+ | 24 | 12.1 |
| HR+/HER2+ | 29 | 14.6 |
| Triple negative | 22 | 11.1 |
| Unknown | 11 | 5.6 |
| Surgery |  |  |
| No surgery | 138 | 69.7 |
| BCS | 12 | 6.1 |
| Mastectomy | 46 | 23.2 |
| Unknown | 2 | 1.0 |
| Extraosseous metastatic sites to lung, liver and brain, No. |  |  |
| 0 | 125 | 63.1 |
| 1 | 63 | 31.8 |
| 2 | 10 | 5.1 |
| Marital status |  |  |
| Married | 136 | 68.7 |
| Unmarried‡ | 57 | 28.8 |
| Unknown† | 5 | 2.5 |
| Abbreviations: BCS, breast conserving surgery; IDC, infiltrating ductal carcinoma; ILC, infiltrating lobular carcinoma; HER2, human epidermal growth factor receptor 2; HR, hormone receptor.  † Including other histology of invasive breast cancer except IDC and ILC.  ‡ Including divorced, separated, single (never married), and widowed. | | |
