# Supplementary material for: Incidence proportions and prognosis of breast cancer patients with bone metastases at initial diagnosis
Source: Cancer Med. 2018 Jul 9;7(8):4156–69. doi: 10.1002/cam4.1668 (PMC6089179; doi:10.1002/cam4.1668)
Supplement: Supplementary file 3 [file CAM4-7-4156-s003.docx]

| **Supplementary Table 3.** Median overall survival of breast cancer patients by extent of systemic metastatic disease | | | | |
| --- | --- | --- | --- | --- |
| **Tumor subtype** | **Type of metastases** | **Overall survival, Median (IQR), mo.** | | ***P*-Value** |
|  |  | **Extraosseous systemic disease only** | **Extraosseous systemic disease and bone metastases** |  |
| HR+/HER2- | Brain | 13.0 (4.0-NR) | 16.0 (6.0-35.0) | 0.738 |
|  | Liver | 29.0 (11.0-NR) | 23.0 (10.0-40.0) | 0.014 |
|  | Lung | 42.0 (14.0-NR) | 31.0 (14.0-NR) | 0.088 |
|  | 2 of 3 | 15.0 (4.0-32.0) | 15.0 (6.0-35.0) | 0.456 |
|  | All 3 | 16.0 (4.0-NR) | 13.0 (3.0–52.0) | 0.968 |
| HR-/HER2+ | Brain | 14.0 (8.0-38.0) | 17.0 (8.0-NR) | 0.573 |
|  | Liver | 46.0 (15.0-NR) | 30.0 (11.0-45.0) | 0.061 |
|  | Lung | 24.0 (11.0-NR) | 21.0 (7.0-NR) | 0.368 |
|  | 2 of 3 | 15.0 (5.0-48.0) | 18.0 (4.0-36.0) | 0.783 |
|  | All 3 | 31.0 (3.0-31.0) | 6.0 (3.0-16.0) | 0.131 |
| HR+/HER2+ | Brain | 30.0 (13.0-NR) | 34.0 (13.0-NR) | 0.679 |
|  | Liver | NR (22.0-NR) | 37.0 (17.0-NR) | 0.037 |
|  | Lung | 55.0 (23.0-NR) | 44.0 (24.0-NR) | 0.631 |
|  | 2 of 3 | 26.0 (5.0-NR) | 23.0 (6.0-NR) | 0.522 |
|  | All 3 | NR (2.0-NR) | 8.0 (2.0-NR) | 0.586 |
| Triple-negative | Brain | 8.0 (3.0-19.0) | 11.0 (3.0-15.0) | 0.853 |
|  | Liver | 14.0 (5.0-23.0) | 8.0 (4.0-13.0) | 0.001 |
|  | Lung | 13.0 (6.0-26.0) | 11.0 (5.0-19.0) | 0.026 |
|  | 2 of 3 | 9.0 (3.0-18.0) | 7.0 (3.0-12.0) | 0.066 |
|  | All 3 | 2.0 (2.0-10.0) | 6.0 (2.0-12.0) | 0.501 |
| Unknown | Brain | 10.0 (5.0-20.0) | 8.0 (2.0-19.0) | 0.657 |
|  | Liver | 16.0 (5.0-39.0) | 14.0 (5.0-29.0) | 0.398 |
|  | Lung | 17.0 (3.0-50.0) | 15.0 (4.0-40.0) | 0.845 |
|  | 2 of 3 | 7.0 (2.0-25.0) | 12.0 (3.0-29.0) | 0.134 |
|  | All 3 | 1.0 (1.0-3.0) | 2.0 (1.0-6.0) | 0.643 |
| All subtypes | Brain | 13.0 (4.0-34.0) | 16.0 (5.0-46.0) | 0.136 |
|  | Liver | 29.0 (11.0-NR) | 22.0 (8.0-43.0) | <0.001 |
|  | Lung | 25.0 (10.0-57.0) | 28.0 (11.0-54.0) | 0.538 |
|  | 2 of 3 | 11.0 (4.0-29.0) | 15.0 (5.0-35.0) | 0.112 |
|  | All 3 | 4.0 (2.0-31.0) | 7.0 (2.0-20.0) | 0.735 |
| Abbreviations: IQR, interquartile range; NR, not reached. | | | | |
